# Supplementary material for: Decreased Survival and Lung Function in Progressive Pulmonary Fibrosis
Source: Medicina (Kaunas). 2023 Feb 5;59(2):296. doi: 10.3390/medicina59020296 (PMC9962949; doi:10.3390/medicina59020296)
Supplement: Supplementary file 1 [file medicina-59-00296-s001.zip › medicina-2136554-supplementary.pdf]

SUPPLEMENTARY DATA

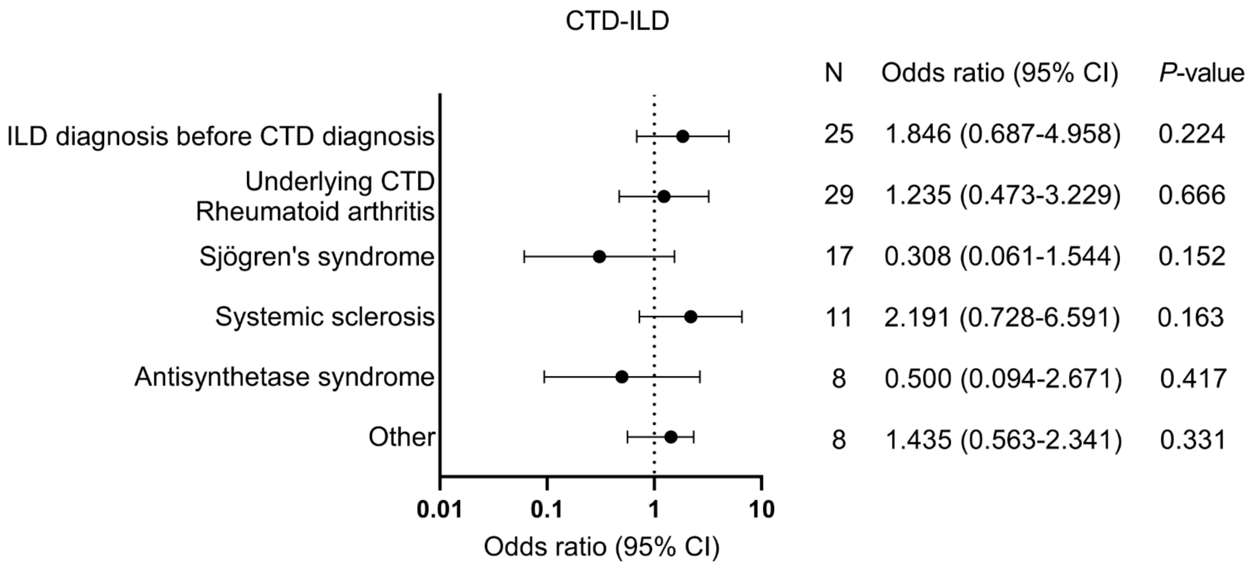

Supplementary Figure S1. Underlying CTD and odds ratio’s for PPF. A.) Odds ratio’s for PPF per CTD subtype and timing of CTD diagnosis (before or after ILD has been demonstrated on radiology scan). CI: Confidence interval, CTD: Connective tissue disease, CTD-ILD: Connective tissue disease-associated interstitial lung disease, and ILD: Interstitial lung disease.
